# Electric field stimulation for tissue engineering applications

**DOI:** 10.1186/s42490-020-00046-0

**Published:** 2021-01-05

**Authors:** Christina N. M. Ryan, Meletios N. Doulgkeroglou, Dimitrios I. Zeugolis

**Affiliations:** 1 Regenerative, Modular & Developmental Engineering Laboratory (REMODEL), National University of Ireland Galway & USI, Galway, Ireland; 2 Science Foundation Ireland (SFI) Centre for Research in Medical Devices (CÚRAM), National University of Ireland Galway, Galway, Ireland; 3 Regenerative, Modular & Developmental Engineering Laboratory (REMODEL), Faculty of Biomedical Sciences, Università della Svizzera Italiana (USI), Lugano, Switzerland

**Keywords:** Electric field, Galvanotaxis, Cell stimulation, Biophysical cues

## Abstract

Electric fields are involved in numerous physiological processes, including directional embryonic development and wound healing following injury. To study these processes in vitro and/or to harness electric field stimulation as a biophysical environmental cue for organised tissue engineering strategies various electric field stimulation systems have been developed. These systems are overall similar in design and have been shown to influence morphology, orientation, migration and phenotype of several different cell types. This review discusses different electric field stimulation setups and their effect on cell response.

## Background

Endogenous electric fields (EFs) are involved in the organisation and development of tissues, as well as in their regeneration following injury [1, 2]. Disruption of endogenous EFs leads to abnormalities [3, 4] and slows down wound healing processes [5]. Physiologically speaking, for example, a polarised epithelium transports ions that maintain a transepithelial potential [6]. When an injury occurs, the transepithelial potential is severely disrupted and an endogenic wound EF occurs that drives epithelial cells to the wound for healing purposes [7]. The magnitude of endogenous EFs varies as a function of species, tissue, location and developmental stage [e.g. 0.02–0.04 V/cm during neocortical activity in ferrets [8]; 0.1–0.2 V/cm in different anatomical parts of axolotl embryos during their developmental stages [9]; 0.42 V/cm in wounded rat corneas [10]; 0.42 V/cm in sliced tips of hindlimb digit of *Notophthalmus viridescens* [11]; 1.1–1.8 V/cm in wounded mouse and human skin [12]; 1–2 V/cm in small skin cuts of cavies [13]; 20–30 mV/cm in mice brain [14].

Considering the importance of EFs in physiological tissue function; disease manifestation and progression; and regeneration, research efforts have been directed towards utilising EFs to study cell response in vitro as a means to better understand the mechanism of action of EF-induced stimulation and develop functional therapeutic interventions. It has now become apparent that EF stimulation in vitro modulates cell morphology, orientation, migration and phenotype commitment, as well as extracellular matrix (ECM) synthesis and orientation [15, 16] and in vivo promotes ECM synthesis [17], modulates ECM deposition [18] and accelerates wound healing [19]. To describe the influence of EF stimulation on cell response, the theories of galvanotaxis (i.e. the process of preferential cell migration towards the anode or the cathode) and galvanotropism (i.e. changes in cell morphology) have been introduced [20, 21]. Over the years, various EF apparati have been used to study the influence of EF stimulation on cell response in vitro with variable degree of complexity and efficiency, jeopardising comprehensive investigation of this in vitro microenvironment modulator. Thus, this review provides and overview of EF setups, describes the function of their most important components and discusses advancements and shortfalls in EF stimulation in controlling cell function.

## Main text

### Electric field cell stimulation setups

**Fig. 1:**
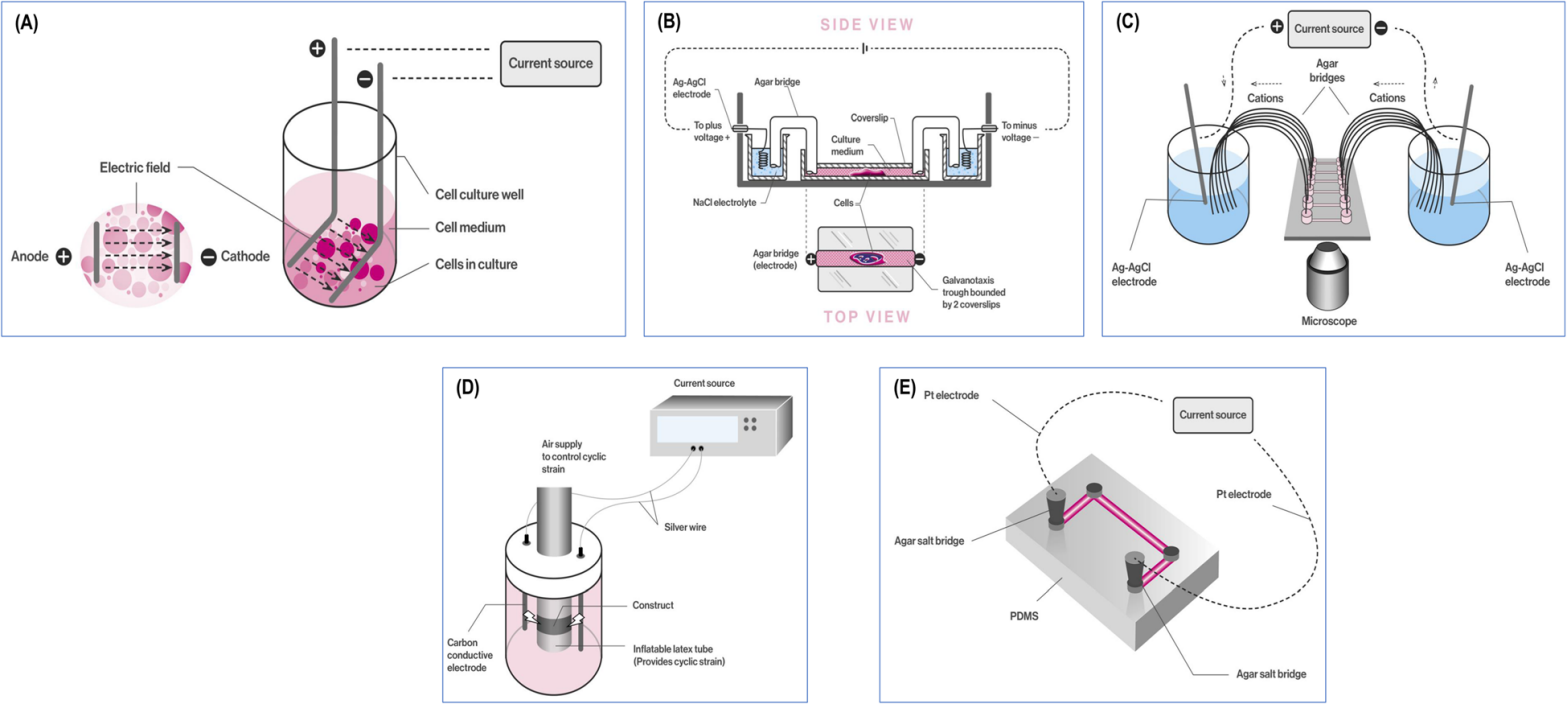
Schematic illustration of various galvanotaxis setups. **a** The simplest setup. **b** The most common setup. **c** Parallel setup that allows multiple experiment simultaneously. **d** Multifactorial setup that allows simultaneous application of electric field stimulation and mechanical loading. **e** Miniaturised, closed system microfluidic setup

In vitro EF stimulation started with a simple setup, where two electrodes were placed at the bottom of a cell culture well and the cells were seeded in between (Fig. 1a). Trial and error experiments (e.g. to avoid media evaporation, avoid electrode degradation products contaminating the cells) have resulted in the current setup, which includes a chamber that contains the media and the cells, with agar bridges transferring the charge from the electrodes immersed into electrolytes to the cell media (Fig. 1b). In the spirit of automation and scalability, parallel setups [22] have been developed that allow for multiple experiments to be conducted simultaneously (Fig. 1c). More complex systems, such as bioreactors capable of combining EF stimulation with mechanical loads [23], have also been developed (Fig. 1d). In the era of miniaturisation, compact, closed-system microfluidic devices (Fig. 1e) that provide more effective control over the uniformity of the EF, mitigate the Joule heating effect, reduce the dimensionality of equipment and offer high data output have also been realised [24, 25].

Independently of the setup, poly(methyl methacrylate) (PMMA) [26–29] and poly(dimethylsiloxane) (PDMS) [30–36] are mostly used for the fabrication of galvanotaxis devices, although some devices have been made from glass [33] or plastic [37]. Further, all systems have a window (usually a glass slide / coverslip), which allows visual assessment of cells before, during and after EF stimulation [38–40]. When chamber size permits, the entire chamber is placed on the stage of an inverted microscope and cell behaviour is observed directly during experiments [41–46]. In the subsequent sections the main components of most EF cell stimulation apparatus are discussed.

### Galvanotaxis chamber

Galvanotaxis chambers are constructed to allow flow of constant electric current directly over the cells within a channel. An early study used a trough that was created by placing two parallel glass coverslips in the centre of a petri dish. The cells were seeded in the created trough and a closed EF was created by connecting the cell culture media with the agar salt bridges to the solution with the electrodes [47]. Due to this simple construction, similar chambers composed of glass slides or coverslips separated by acetate or silicon spacers and held together with silicone grease or adhesive have been fabricated [48–54]. To reduce time, effort and costs associated with continuous chamber fabrication, a modular chamber design comprised of parallel plates that allow glass slides or coverslips plated with cells to be inserted and removed at ease without affecting the chamber structure have been developed using various materials (e.g. plexiglass, polycarbonate, acrylic, graphene and PMMA) [55–61]. PDMS is featured in several setups either as a primary material from which chambers may be excised [62] or due to its insulating properties that allow independent electrical stimulation of rows of wells [63]. Further, its versatility of stiffness modification [64], allows for simultaneous assessment of substrate rigidity and EF stimulation on cell response. To assist cell adhesion, surfaces used as channels for cell seeding are often coated with ECM proteins (e.g. laminin, fibronectin, collagen) [65–70] and to improve cell motility and alignment, microgrooves are etched onto glass / quartz slides [71–73].

### Electrodes

Electric current is generally passed through the galvanotaxis chamber by placing electrodes into phosphate buffered saline (PBS) or Steinberg’s solution reservoirs, from which agarose salt bridges form a conducting pathway to the chamber with the cathode connected at one side of the chamber and the anode to the other [46, 47]. Conductive bridges, composed of plastic or glass tubing are filled with agarose (2–4%) and can be of different lengths [from 6 cm [74, 75] to 35 cm [55, 59], although most setups incorporate bridges of 15 to 20 cm [76–78]]. Some groups have even bent tissue culture pipettes into U-shapes and used them as agar-salt bridges, which have the added advantage of already being sterile [79]. Systems with reduced size agar bridges embedded within the galvanotaxis chamber [35, 36] or even setups without salt bridges, which facilitate the design of reduced size devices [80] have also been reported, albeit not extensively. The bridges also act as safeguards to reduce heat exposure of cells via Joule heating of the chamber [75] and prevent electrolysis products (e.g. metal ions) [81] produced at the electrodes from contaminating cells within the chamber [82]. Aluminium [83], carbon [84, 85], copper [86, 87], platinum [60, 88] and stainless-steel [89, 90] have been used as electrodes across a range of direct current (DC) EF stimulation systems, however silver-silver chloride (Ag/AgCl) electrodes are the most commonly used [46, 47]. These are favoured as the only species involved in the electrochemical reactions at the electrode surface are chloride ions, thus eliminating unwanted reactions associated with electrodes, such as platinum [91]. They convert electron flow to a chloride ion flow from the cathode to the anode through the conducting pathways. Ag/AgCl electrodes can be fabricated from silver wire by soaking for up to 1 h in a hypochlorite / bleach solution, or in 1 M HCl and then chloridised for 30 min at a current of 5–10 mA cm^2^ [59, 91]. These electrodes can then be stored in distilled water or PBS for several weeks. In some setups, the electrodes have been integrated into the galvanotaxis chamber by coiling them about 5 cm into agarose embedded within the platform [91]. This saving in size of the setup allows the platform to be efficiently placed within a live cell chamber, whereby humidity, CO_2_ partial pressure and temperature can be controlled relatively ease.

### Power supply and electric field stimulation regimes

EF stimulation utilises either DC or alternating current (AC). DC is a steady mono-flow / unidirectional current, whereas AC has a sinusoidal form and constantly switches direction. As in the extracellular space of plants and animals, DC signals are primarily observed [92], the vast majority of EF cell stimulation studies use DC. Nonetheless, AC has also been selected to either compare its effect with the frequently used DC stimulation [93], or to recreate physiological EFs, in the case of the central nervous system that neurons are exposed to oscillating endogenous EFs [94, 95]. Over the years, numerous cell types have been exposed to different EF strengths (0–10 V/cm) and stimulation duration (0–72 h) (Table 1). To achieve the required EF strength, DC power supplies (e.g. Keithley SourceMeter®) have been used that work with DC currents of 0.0–0.3 mA and generate EFs of 0–6 V/cm [97, 98]. Eight-channel programmable power simulators (e.g. Master-8, AMPI) [61] generating EFs up to 4.5 V/cm, multi-potentiostats (e.g. CH1040A, CH Instruments) generating EFs of 0.1 V/cm and currents of 0.0–0.1 mA [86] and the commonly found in laboratory setups gel electrophoresis (e.g. FB600, Thermo Fisher Scientific) power sources [58, 99] have also been used with an EF range of 0–10 V/cm. For the measurement and adjustment of current and field strength during a stimulation, multi-meters can be positioned respectively in series and in parallel with the chamber [55]. In addition, current density and correlating EFs have been altered not only by adjusting applied current or voltage, but also by altering resistance through the channel by varying the channel widths (0.5–3.0 cm) [86]. For the application of AC EFs, function (waveform) generators that provide both type of currents may be used (e.g. Precision 4011A, PASCO Scientific) [93] with the AC component ranging less than the regimes observed in DC, usually within 0–1 V/cm [58, 100].

**Table 1:** Indicative examples of the influence of electric field stimulation in various human cell types in vitro and in vivo

Cell type	Power Supply	Electric Field Strength (V/cm)	Stimulation Duration (h)	Preferred Direction	Major Result
Chondrocytes	DC, Keithley Instruments (USA)	6	3	Bidirectional (dependent on passage of cells)	EF directed migration was influenced by passage [27]
Keratinocytes	DC & AC PASCO Scientific (USA)	0.4 at 1.6 or 160 Hz (AC) / 1 (DC)	1	Cathode	Verification of electromechanical model for migration [93]
Mammary epithelial cells	DC, Pine (USA)	0.13–1.0	6	Anode	Clustered cells were more sensitive to alignment, but migrated slower than isolated cells [83]
Osteoblasts	DC, Biometra (Germany)	0.15–0.45	7	Anode	Upregulation of ion channel gene, associating Ca^2+^ with migration speed [96]
Peripheral blood lymphocytes	DC, Agilent Technologies (USA)	0.15–2	0.5–2.0	Cathode	Directed migration in vitro and in vivo and activated intracellular kinase pathways [37]
Neuroblastoma cells	DC, AMPI (Israel)	0.045–4.5	4	Anode	Enhancement of cell mobility [61]
Bone marrow stem cells	DC, Glassman FC (USA)	0.2–5	15	Cathode	Donor did not influence migration direction and morphological changes but affected response time to EF, migration speed and cell viability [22]

### Generated forces during a galvanotaxis experiment

When a cell migrates in any substrate, its displacement gives rise to three-dimensional tractional forces [101], which is also the case for EF assisted migration. During EF stimulation, cells are exposed to forces from the EF itself and from the culture substrate. The stress can be perpendicular and horizontal to the direction of the EF. Forces also develop between the surfaces of the cells, as they touch each other in the restricted space of a galvanotaxis chamber during a collective migration. The interaction of the cells leads to a parallel to the direction shear stress and a perpendicular to the direction normal stress [102]. It has been shown that by the onset of EF in a keratinocyte monolayer [103], the intercellular stress component in the perpendicular axis to the EF direction increases significantly in comparison to the stress component in parallel to the EF direction and that migration is independent of the reorientation of the intercellular stress. In addition, the flow, which can be hypothesised as laminar, applies hydrodynamic forces to the cells. These forces can be calculated by the hydrodynamic equation of laminar flow mechanics.

The exact mechanism regarding galvanotaxis-induced motility is still unclear. In literature, different hypotheses have been formulated regarding the decisive factor for cell migration during galvanotaxis. These hypotheses include the effect of flow, due to hydrodynamic cell forces, on the cell membrane [104]; the activation of electrotaxis, owed to change of cell membrane polarity, which in turn is driven by an asymmetric local concentration of ions [105]; and the electrophoresis of charged membrane components (e.g. proteins) [106, 107]. The normally occurring hydrodynamic forces alone have not been proven to contribute to directional migration, since cells were observed to move randomly in the absence of an EF in almost all the reported experiments [54, 103] However, when an external shear stress stimuli was applied, migration was retained in the preferential direction even without the application of an EF [35]. A recent work investigated the role of integrins by testing hamster ovary modified cell lines that express specific human integrins and concluded that different subsets of integrins may promote normal or reverse directional migration during galvanotaxis, thus highlighting the importance of the intracellular domain with cell migration [108]. It should be noted that the strength of the EF increases the aligned directed locomotion of the cells, as it has been shown in numerical simulations [109] and experimental data [110, 111]. However, differences were observed [83] in the time of response and the required EF intensity needed to trigger migration for clustered and isolated cells. It should be noted that according to the cell type, cells may show different preferences in anodal or cathodal directed migration (Table 1).

### Electric field stimulation in vitro and in vivo

Although the influence of DC and AC EFs on cell response in vitro and in vivo has been the subject of many investigations (Table 1), it is worth noting that most studies focus on the alignment and migration patterns that DC EFs induce to cells and only a few studies have assessed the influence of EFs on cellular functions in vitro and tissue response in vivo. In general, subject to the cell population, DC EF of up to 10 V/cm and for up to 72 h are efficient in controlling cell orientation and migration [71, 72], increase cell proliferation [112, 113]; and do not affect cell metabolic activity and viability [114–117]. Stem cell differentiation has also been studied; for example, DC EFs of 0.1–1.0 V/cm [118, 119] and pulsed DC EFs of 50 Hz and 6 V/cm peak-to-peak amplitude for 6 h per day [120] have been shown to favour osteogenic differentiation.

With respect to AC EF stimulation, although it has been shown to affect cellular functions, alone has not been shown consistently to result in controlled cell orientation and migration. For example, AC EFs of 10 Hz and 50 Hz have been shown to sustain a more immature phenotype in porcine neural progenitor cells, without promoting alignment and affecting proliferation [100]. AC EF stimulation (20 mV/cm, 60 kHz, 40 min per day for 20 days) has also been shown to not affect cell morphology and metabolic activity in human stem cell cultures and to increase osteogenic differentiation [121]. Regarding differentiation, AC EFs have been used for both osteogenic [122–124] and chondrogenic [125, 126] differentiation of stem cells. When mouse neural stem cells were encapsulated in alginate hydrogel beads and subjected to AC EFs (0.1 to 10 Hz; 2, 4, 16 V/m; 14 and 21 days), it was reported that 1 Hz frequency enhanced viability, whilst differentiation was promoted or inhibited subject to culture time and EF frequency (cell morphology analysis was not conducted) [127].

When DC was directly compared to AC in rat neural stem/progenitor cell cultures, it was found that differentiation and migration were enhanced and viability was decreased in DC EFs, whilst AC EF had no effect [58]. Interestingly, in human keratinocytes isolated from neonatal foreskin cultures, AC led to random migration; DC alone and DC combined with AC resulted in cathodal direction; and DC combined with 160 Hz AC resulted in enhanced migration in comparison to DC alone and DC combined with 1.6 Hz AC [93]. Other than cell morphology and migration analysis studies, more in depth biological analysis studies are required to clearly illustrate whether there are any beneficial effects in combing DC with AC EF stimulation.

In in vivo setting, preliminary studies advocate the use of EF stimulation. For example, the migration of human peripheral blood lymphocytes was enhanced in mouse ear skin model when an external EF was applied [37]. EFs have also been shown to promote migration and differentiation of neural progenitor cells in a rat model of chronic-phase ischemic stroke [128]. In a similar manner, electrodes were inserted in a rat brain and stimulated transplanted human neural progenitor cells, resulting in directed migration and increased motility [129]. Furthermore, transvaginal electric stimulation in female mice has shown activation and proliferation of fibroblasts [130].

In clinical setting, electric stimulation has been used in different instances with mixed outcomes. Recent studies, for example, include the use of electric stimulation to treat neurogenic bowel dysfunction in patients that suffered spinal cord injuries, but without consistent results [131]. On the other hand, EF stimulation resulted in a reliable recovery of motor functions in patients experienced a stroke [132], an improvement in visual abilities by the placement skin electrodes in patients with retinitis pigmentosa [133] and accelerated wound healing [19], collectively indicating the potential of EF stimulation in reparative medicine.

## Conclusions

Electric field stimulation is continuously gaining pace as a means to control cell orientation, migration and phenotype in vitro and in vivo. Direct current electric fields (up to 10 V/cm) are favoured among investigators, as such signals are primarily encountered in the extracellular space of plants and animals. Although variable in complexity galvanotaxis chambers have been used over the years, the most popular setups are comprised of glass slides for cell seeding, transparent polymers that allow real-time cell visualisation, Ag/AgCl electrodes that eliminate toxic electrode degradation products and agarose salt bridges in phosphate buffered saline to prevent them from drying and to stabilise electrode potentials. It is worth noting that despite the promising in vitro data, only a few studies have assessed the influence of electric field stimulation in vivo and in clinical setting. Standardisation and automation of the processes will allow more intense investigation of electric field stimulation in the years to come.

## Data Availability

Not applicable.

## Abbreviations

AC: Alternating current
Ag/AgCl: Silver-silver chloride
DC: Direct current
EF: Electric field
ECM: Extracellular matrix
PBS: Phosphate buffered saline
PDMS: Poly(dimethylsiloxane)
PMMA: Poly(methylmethacrylate)
